# Fluorescence Lifetime Imaging Microscopy, a Novel Diagnostic Tool for Metastatic Cell Detection in the Cerebrospinal Fluid of Children with Medulloblastoma

**DOI:** 10.1038/s41598-017-03892-6

**Published:** 2017-06-16

**Authors:** Sivan Gershanov, Shalom Michowiz, Helen Toledano, Gilad Yahav, Orit Barinfeld, Avraham Hirshberg, Haim Ben-Zvi, Gabriel Mircus, Mali Salmon-Divon, Dror Fixler, Nitza Goldenberg-Cohen

**Affiliations:** 1 Genomic Bioinformatics Laboratory, Department of Molecular Biology, Ariel University, Ariel, 40700 Israel; 2 The Krieger Eye Research Laboratory, Felsenstein Medical Research Center, Beilinson Hospital, Petach Tikva 4941492, affiliated to Tel Aviv University, Tel Aviv, 6997801 Israel; 3 Faculty of Engineering and Institute of Nanotechnology and Advanced Materials, Bar Ilan University, Ramat Gan, 5290002 Israel; 4 Department of Pediatric Neurosurgery, Schneider Children’s Medical Center of Israel, Petach Tikva, 4920235 Israel; 5 Department of Pediatric Oncology, Schneider Children’s Medical Center of Israel, Petach Tikva, 4920235 Israel; 6 Department of Ophthalmology, Bnai Zion Medical Center, Haifa, 3339419 Israel; 7 Laboratory of Microbiology, Rabin Medical Center – Beilinson Hospital, Petach Tikva, 4941492 Israel; 8 Sackler Faculty of Medicine, Tel Aviv University, Tel Aviv, 6997801 Israel; 9 Department of Oral Pathology and Oral Medicine, Maurice and Gabriela Goldschleger School of Dental Medicine, Tel Aviv University, Tel Aviv, 6997801 Israel

## Abstract

In pediatric brain tumours, dissemination of malignant cells within the central nervous system confers poor prognosis and determines treatment intensity, but is often undetectable by imaging or cytology. This study describes the use of fluorescence lifetime (FLT) imaging microscopy (FLIM), a novel diagnostic tool, for detection of metastatic spread. The study group included 15 children with medulloblastoma and 2 with atypical teratoid/rhabdoid tumour. Cells extracted from the tumour and the cerebrospinal fluid (CSF) 2 weeks postoperatively and repeatedly during chemo/radiotherapy were subjected to nuclear staining followed by FLT measurement and cytological study. Control CSF samples were collected from patients with infectious/inflammatory disease attending the same hospital. Median FLT was prolonged in tumour cells (4.27 ± 0.28 ns; *P* < 2.2*10^−16^) and CSF metastatic cells obtained before chemo/radiotherapy (6.28 ± 0.22 ns; *P* < 2.2*10^−16^); normal in inflammatory control cells (2.6 ± 0.04 ns) and cells from children without metastasis before chemo/radiotherapy (2.62 ± 0.23 ns; *P* = 0.858) and following treatment (2.62 ± 0.21 ns; *P* = 0.053); and short in CSF metastatic cells obtained after chemo/radiotherapy (2.40 ± 0.2 ns; *P* < 2.2*10^−16^). FLIM is a simple test that can potentially identify CSF spread of brain tumours. FLT changes in accordance with treatment, with significant prolonged median values in tumours and metastases. More accurate detection of metastatic cells may guide personalised treatment and improve the therapeutic outcome.

## Introduction

Brain tumours account for at least one-fourth of all cases of cancer in the pediatric age group and are the leading cause of cancer-related death in this population^1, 2^. Embryonal tumours are the second most common type after gliomas. Three-fourths of embryonal tumors are medulloblastomas^3^, making medulloblastoma the most common malignant tumour of childhood (average age at diagnosis, 6 years).

Medulloblastomas affect the posterior fossa^4^, and one-third of patients have metastatic spread already at diagnosis. Treatment consists of surgical resection, craniospinal irradiation, and aggressive chemotherapy sometimes supplemented by autologous bone-marrow transplantation. The 5-year overall survival rate ranges from 60% to 70%^5^. Despite recent advances in treatment, 30% to 40% of children experience tumour recurrence, and most of them die from disease especially if previously irradiated^6^.

Another highly malignant tumour of childhood is atypical teratoid/rhabdoid tumour (ATRT), usually diagnosed in the first 2 years of life^7^. Although ATRT appears to be chemosensitive, chemotherapy-only regimens are often insufficient^8, 9^, and there are few long-term survivors. Median overall survival is around 10 months^10^, and with chemotherapy^11, 12^, radiotherapy^13^, or both^14, 15^, it may reach 14–18 months or more.

Clinically, malignant brain tumours usually metastasize within the central nervous system (CNS) and very rarely to extraneural sites. The dissemination of malignant cells within the CNS is associated with a very low survival rate^16^. However, current means of detection of CNS disease, mainly preoperative imaging and postoperative cytologic study of the cerebrospinal fluid (CSF), are limited and reviewer-dependent. This is crucial, because diagnostic errors may lead to under-treatment, which may be life-threatening, or over-treatment, which places patients at unnecessary risk of severe adverse effects and future disability. Therefore, novel diagnostic solutions are urgently needed.

Studies of light propagation in tissue models have shown that different tissues have different diffusion reflection profiles and that light propagates differently through different cell types^17^. These factors may help clinicians differentiate malignant from non-malignant cells. Fluorescence lifetime imaging microscopy (FLIM) is a novel spectroscopic technique that incorporates hitherto uncorrelated mathematical^18^ and physical^19^ methods of light-tissue interaction^20, 21^. FLIM produces images based on differences in the rate of fluorescence decay, providing both temporal and spatial information on changes in the fluorescence lifetime (FLT) of fluorescently labelled components in a sample. Contrast is generated by the FLT of individual fluorophores at each pixel. FLIM has a wide range of biological and biomedical applications^22–24^, such as quantification of tissue morphology and as an add-on in high-density protein arrays^25^. It differs from the earlier steady-state fluorescence techniques used in the bio-sciences which are not useful for quantitative investigation of cellular function at the molecular level.

The aim of the present study was to determine if FLIM technology may be used to detect and monitor metastatic cells in the CSF in children with brain tumours. The early detection of metastasis in the CSF has important implications for improving treatment regimes, monitoring of disease progression, identification of disease recurrence, and assessment of the success of a selected therapy.

## Results

Seventeen children were included in the study group: 15 with medulloblastoma and 2 with ATRT. Their demographic, clinical, and treatment data are shown in Table 1. Mean age at diagnosis was 5.2 ± 3.2 years. Ten patients had localised disease at diagnosis and 7 had metastases according to imaging, biopsy, or CSF cytology findings (Table 1). The maximum duration of CSF follow-up was 21 months from diagnosis.

**Table 1:** Demographic and clinical data of the study group.

Pt. no.	Sex	Age (yr)*	Diagnosis	Localised/Metastatic	Relapse^†^	Chemo-therapy	Radio-therapy	BMT
1	M	3	Desmo-plastic MB	Localised	−	+	−	+
2	F	6	Classical MB	Localised	−	+	+	−
3	M	4	Classical MB	Localised	−	+	+	−
4	M	10	Classical MB	Localised	−	−	+	−
5	M	11	Classical MB	Localised	−	+	+	−
6	F	12	Classical MB	Localised	−	+	+	+
7	M	1	Desmo-plastic MB	Localised	−	+	−	−
8	F	2	Classical MB	Metastatic	−	+	+	+
9	F	4	Classical MB	Metastatic	−	+	+	+
10	M	0.5	ATRT	Metastatic	+	+	+	−
11	F	4	Classical MB	Localised	−	+	+	−
12	M	4	Classical MB	Localised	−	+	+	−
13	M	5	Classical MB	Metastatic	−	+	+	−
14	F	6	Classical MB	Metastatic	−	+	+	−
15	F	8	Desmo-plastic MB	Metastatic	−	+	+	+
16	F	5	Classical MB	Metastatic	−	+	+	+
17	F	3	ATRT	Localised	−	+	+	−

^*^At diagnosis.

^†^As of completion of this study.

BMT, bone marrow transplantation, MB, medulloblastoma, ATRT, atypical teratoid/rhabdoid tumour.

**Figure 1:**
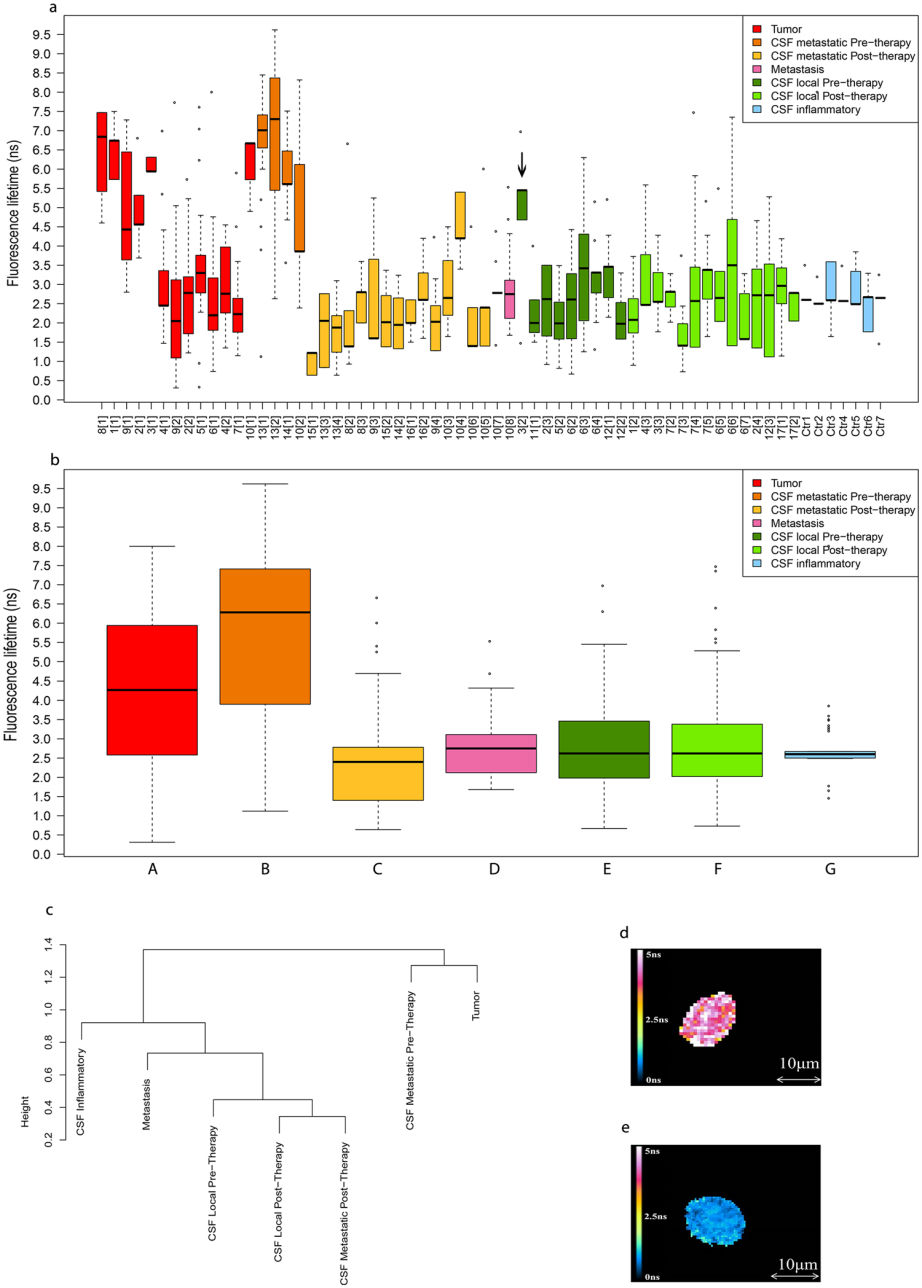
(**a**) Distribution of the FLT (ns) measured for each slide. Patients were categorised according to stage of treatment. The control group contains slides from patient with inflammatory disease. (**b**) Distribution of the fluorescence lifetime (ns) in each source group. (**c**) Hierarchical clustering (average, 1-correlation) for each source group of patients. (Patient numbers and groups are keyed to Tables 1 and 2 respectively). An image of a cell produced by the FLIM system (**d**) from a group B patient with a FLT of 4.8 ± 0.43 ns, and (**e**) from a group C patient with a FLT of 1.17 ± 0.3 ns.

Findings on FLIM study in the individual patients are shown in Table 2 and Fig. 1a. Median and median absolute deviation FLT values were measured to overcome the variability of each slide. In addition, they are insensitive to the presence of outliers, unlike the standard mean/standard deviation combination. Median FLT in cells originating from the tumour, calculated per slide per patient, ranged from 2.05 to 6.84 ns. For the CSF measurement, at least 1–2 repeated samples were obtained from all children, including 5 children in whom FLT was measured in at least 3 samples, for a total of 43 samples. Median FLT values in the CSF were as follows: one sample obtained at diagnosis (surgery), 2.62 ns; 13 samples obtained before chemo/radiotherapy (all but one less than one month from diagnosis), 1.98–7.3 ns; 20 samples obtained during chemo/radiotherapy, 1.4–4.2 ns; and 10 samples obtained after chemo/radiotherapy, 1.22–2.965 ns. One patient (no.10) was found to have a relapse in the lumbar spinal cord 16 months after initial diagnosis, the median FLT in cells obtained at biopsy of the metastasis was 2.75 ns (range 1.68–5.53 ns). One patient (no. 8) was found to have disseminated metastasis on diagnostic magnetic resonance imaging study. She did not undergo repeated CSF sampling because it was considered unethical given the radiology findings.

**Table 2:** FLT of each slide, obtained using FILM measurements, per patient categorised according to stage of treatment (at diagnosis, before chemo/radiotherapy, during chemo/radiotherapy, after chemo/radiotherapy) and per patient of control group: inflammatory disease.

Pt. no. [no. of sample] Loc/Met	Source	FLT range (ns)	FLT median (ns)	No. cells/slide	Time from surgery (mo)
**At diagnosis (surgery)**					
1 [1] Loc	Tumour	5.73–7.5	6.74	11	
2 [1] Loc	Tumour	3.69–6.8	4.56	33	
2 [2] Loc	Tumour	1.22–5.73	2.78	106	
3 [1] Loc	Tumour	5.94–6.31	5.94	5	
4 [1] Loc	Tumour	1.47–6.99	2.45	52	
4 [2] Loc	Tumour	1.35–4.55	2.76	39	
5 [1] Loc	Tumour	0.33–7.61	3.3	79	
6 [1] Loc	Tumour	0.74–8	2.2	66	
7 [1] Loc	Tumour	1.15–5.9	2.23	46	
8 [1] Met	Tumour	4.6–7.47	6.84	12	
9 [1] Met	Tumour	2.8–7.28	4.43	55	
9 [2] Met	Tumour	0.31–7.7	2.05	93	
10 [1] Met	Tumour	4.9–6.67	6.67	16	
**Before chemo/radiotherapy (up to 1 mo. from diagnosis)**					
2 [3] Loc	CSF	0.92–3.5	2.62	27	<1
3 [2] Loc	CSF	1.47–6.97	5.45	5	<1
5 [2] Loc	CSF	0.82–3.49	1.99	22	<1
6 [2] Loc	CSF	0.67–4.43	2.61	27	<1
6 [3] Loc	CSF	1.25–6.3	3.42	45	<1
6 [4] Loc	CSF	1.31–5.15	3.31	25	<1
10 [2] Met	CSF	2.39–8.32	3.86	25	<1
11 [1] Loc	CSF	1.5–4	2	15	<1
12 [1] Loc	CSF	2.15–5.21	3.46	37	<1
12 [2] Loc	CSF	1.58–3.3	1.98	30	<1
13 [1] Met	CSF	1.12–8.45	7.01	25	<1
13 [2] Met	CSF	2.63–9.62	7.3	14	<1
14 [1] Met	CSF	3.56–7.51	5.61	35	2
**During chemo/radiotherapy**					
1 [2] Loc	CSF	0.895–3.73	2.075	9	21
2 [4] Loc	CSF	1.35–4.66	2.72	48	6
4 [3] Loc	CSF	2.47–5.59	2.47	27	1
6 [5] Loc	CSF	2.04–5.49	2.65	47	1.5
6 [6] Loc	CSF	1.41–7.35	3.5	39	2
6 [7] Loc	CSF	1.58–3.28	1.58	42	4
7 [2] Loc	CSF	2.02–3.28	2.81	43	1
7 [3] Loc	CSF	0.73–3.75	1.41	40	4
7 [4] Loc	CSF	1.37–7.47	2.57	73	5
7 [5] Loc	CSF	1.65–5.16	3.38	82	6
10 [3] Met	CSF	1.65–4.5	2.65	10	3
10 [4] Met	CSF	3.4–5.4	4.2	20	5
10 [5] Met	CSF	1.4–6	2.4	25	6
10 [6] Met	CSF	1.4–4.5	1.4	25	7
10 [7] Met	CSF	1.42–4.38	2.78	46	11
12 [3] Loc	CSF	1.12–5.28	2.72	49	2
13 [3] Met	CSF	0.84–2.76	2.055	8	1
13 [4] Met	CSF	0.64–3.1	1.88	7	1
16 [1] Met	CSF	1.5–2.6	2	13	1
16 [2] Met	CSF	1.6–4.2	2.6	28	4
**After chemo/radiotherapy**					
3 [3] Loc	CSF	1.77–4.28	2.55	25	10
8 [2]* Met	CSF	0.93–6.66	1.39	7	7
8 [3] Met	CSF	2–3.6	2.8	20	13
9 [3] Met	CSF	1.6–5.25	1.6	19	3
9 [4] Met	CSF	1.28–4.23	2.03	27	7
14 [2] Met	CSF	1.33–3.24	1.95	53	9
15 [1] Met	CSF	0.635–1.22	1.22	5	8
15 [2]^†^ Met	CSF	1.38–3.37	2.02	25	15
17 [1] Loc	CSF	1.14–4.19	2.965	50	11
17 [2] Loc	CSF	2.05–2.78	2.78	30	13
**Metastasis**					
10 [8] Met	Tumour spinal cord	1.68–5.53	2.75	61	16
**Control group: Inflammatory disease**					
Ctr 1	CSF	2.6–3.5	2.6	11	
Ctr 2	CSF	2.5–3.2 (8)^#^	2.5	28	
Ctr 3	CSF	1.65–3.59	2.59	16	
Ctr 4	CSF	2.57–3.48	2.57	16	
Ctr 5	CSF	2.49–3.85	2.49	14	
Ctr 6	CSF	1.77–3.29	2.67	36	
Ctr 7	CSF	1.45–3.25	2.65	31	

^*^After chemotherapy, before radiation.

^†^Six months after completion of radiation.

^#^Only one cell out of 28 examined was high as 8.

FLT, fluorescence lifetime, CSF, cerebrospinal fluid, Loc, localised, Met, metastatic.

In most cases, the FLT value was higher at diagnosis than at the end of follow-up.

Seven CSF samples were collected from patients with a noncancerous diagnosis of infection/inflammation. Median FLT, calculated per slide per patient (11–36 cells per slide) ranged from 2.49 to 2.67 ns (Table 2 and Fig. 1a).

The FLIM results are summarized in Table 3 and Fig. 1b,c. The slides were grouped according to source: tumour, group A; CSF, with further breakdown by spread at diagnosis (metastatic/local) and stage of therapy (before/after), groups B, C, E, F; metastasis (group D) (one slide of metastasis in the lumbar spinal cord, patient 10); and non-oncogenic, inflammatory disease (control), group G. FLT values were categorized as prolonged, normal/medium, and short. Every study group (A-F) was individually compared with the control group (G), and groups A, C, D, E, and F were compared against the CSF metastatic, pre-therapy group B (Table 3).

**Table 3:** Statistical data (after normalization) of the distribution of fluorescence lifetime (ns) in each source group.

Group	Source (no. of cells)	Min.	1st Qu.	Median	Mean	3rd Qu.	Max.	*P* value against Control	*P* value against group B
A	Tumour (n = 613)	0.31	2.45	4.27	4.21	5.94	8.00	<2.2*10^−16^	<2.2*10^−16^
B	CSF metastatic, pre-therapy (n = 99)	1.12	3.90	6.28	6.02	7.41	9.62	<2.2*10^−16^	
C	CSF metastatic, post-therapy^†^ (n = 258)	0.64	1.40	2.40	2.40	2.78	6.66	<2.2*10^−16^	<2.2*10^−16^
D	Metastasis (n = 61)	1.68	2.12	2.75	2.88	3.11	5.53	0.015	<2.2*10^−16^
E	CSF local, pre-therapy (n = 229)	0.67	1.98	2.62	2.87	3.46	6.97	0.858	<2.2*10^−16^
F	CSF local, post-therapy^†^ (n = 604)	0.73	2.02	2.62	2.70	3.38	7.47	0.053	<2.2*10^−16^
	**Control group: Inflammatory disease**								
G	CSF Inflammatory (n = 152)	1.45	2.50	2.60	2.81	2.67	8.00*		<2.2*10^−16^

Slides were combined as a group according to the source (tumour/CSF/metastasis), spread at diagnosis (localised/metastatic) and stage of therapy (pre-therapy/post-therapy^†^). Control group contains slides from patient with inflammatory disease. We used Wilcoxon signed rank test to compared control group against every single group separately (*P* value against control group). We also compared every single group against the CSF metastatic, pre-therapy group (*P* value against group B).

*One cell.

^†^During and after chemo/radiotherapy.

ATRT, atypical teratoid/rhabdoid tumour, CSF, cerebrospinal fluid.

Prolonged FLT values were seen in both the original tumour cells (group A; median 4.27 ± 0.28 ns, range 0.31–8.00 ns; *P < *2.2*10^−16^) and metastatic CSF cells before treatment (group B; median 6.28 ± 0.22 ns, range 1.12–9.62 ns; *P* < 2.2*10^−16^). Group B had a statistically significantly longer FLT than every other group. Normal/medium FLT values were seen in CSF cells obtained from children with non-metastatic disease before chemo/radiotherapy (group E; median 2.62 ± 0.23 ns, range 0.67–6.97 ns; *P* = 0.858) and after chemo/radiotherapy (group F; median 2.62 ± 0.21 ns, range 0.73–7.47 ns; *P* = 0.053). Short FLT values were seen in CSF cells from children with metastases after chemo/radiotherapy (group C, median 2.40 ± 0.2 ns, range 0.635–6.66 ns; *P* < 2.2*10^−16^). Figure 1d,e demonstrates the image produced by the FLIM system for each cell. CSF cells from the patients with non-oncologic inflammatory disease (control group G) had normal/medium FLT values (median 2.6 ± 0.04 ns, range 1.45–3.85 ns). Of note, there was only case of extreme FLT (8 ns), in 1 of 28 cells from the same slide and of the total 152 cells (Tables 2 and 3).

Figure 1a shows the results of all slides (tumour cell samples or repeated CSF samples). One CSF sample from patient 3 showing clinically localized disease before treatment had extremely high FLT values relative to the other samples (Fig. 1a, black arrow), probably indicating occult, clinically undetected metastases.

**Figure 2:**
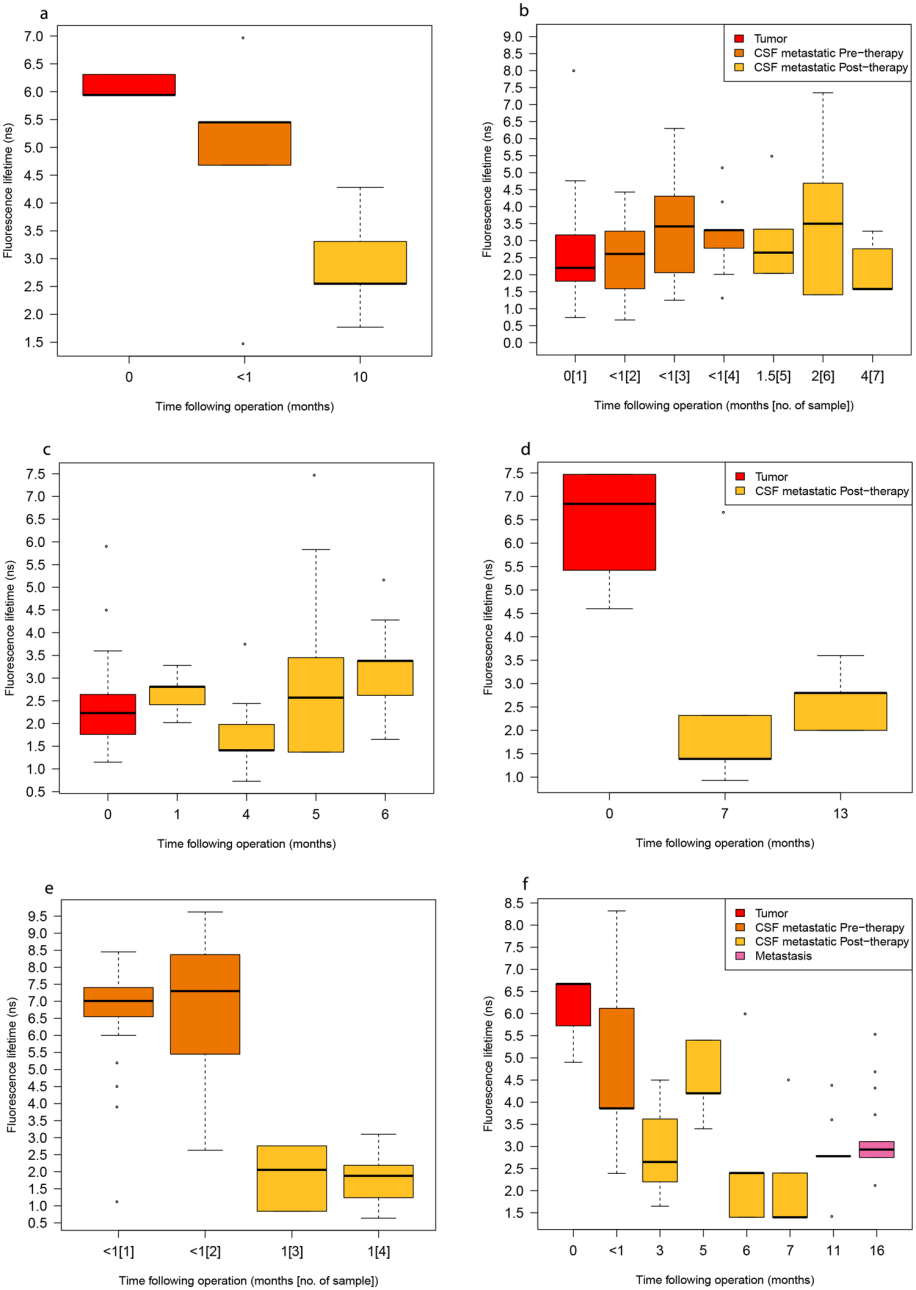
Fluorescence lifetime for each stage of therapy in 5 patients with medulloblastoma, (**a**) patient 3, (**b**) patient 6, (**c**) patient 7, (**d**) patient 8, (**e**) patient 13, and one patient with ATRT (**f**), patient 10. (Patient numbers are keyed to Tables 1 and 2).

The findings in samples from 5 children with medulloblastoma are shown separately in Fig. 2(a–e). There was a significant difference in FLT between the tumour and the CSF samples after chemo/radiotherapy. Figure 2f shows the findings in a child with ATRT who underwent CSF sampling 6 times during treatment and was later found to have a distant metastasis in the lumbar spinal cord.

**Figure 3:**
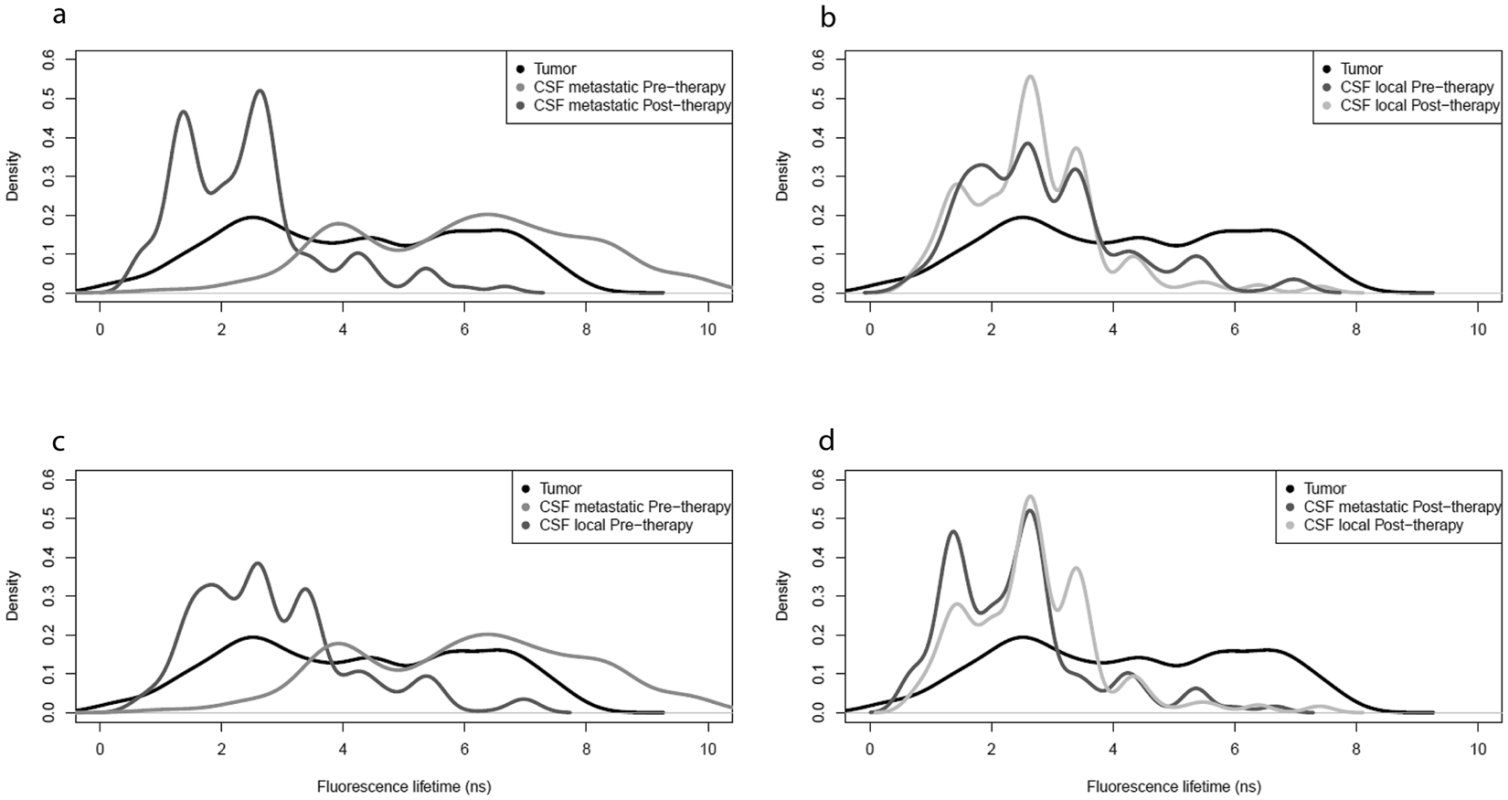
Density plots of fluorescence lifetime findings in tumour cells and CSF cells from patients with (**a**) metastatic disease, (**b**) localised disease (**c**), before chemo/radiotherapy (**d**), after chemo/radiotherapy.

To clarify the results, the data were entered into a density plot (Fig. 3). We identified a population of tumour cells with localised or metastatic spread at diagnosis with high FLT values (>3.5 ns), in addition to a large population of CSF cells from children with localised disease before chemo/radiation, with shorter FLT than the tumour sample, probably indicating reactive (and not tumour) cells. The CSF samples from children with metastatic medulloblastoma before chemo/radiation showed one cell population with a similar FLT to the tumour sample (high) and a second population with a similarly low FLT to the localized disease CSF samples, probably indicating a mix of malignant and reactive cells.

## Discussion

This study is the first to describe the use of the novel FLIM system to differentiate inflammatory from metastatic cells in the CSF of children with malignant brain tumours. Furthermore, the technique enabled us to distinguish localized disease (no spread of malignant cells in the CSF) from metastatic disease at diagnosis. The ability to detect occult metastasis at diagnosis using a simple CSF examination has important therapeutic implications. FLIM also makes disease follow-up possible with repeated CSF sampling for early detection of recurrence.

We found 3 different FLT measurements in CSF cells, clearly differentiating normal inflammatory non-malignant cells from malignant cells. The median FLT of metastatic CSF samples was significantly longer than that of the other CSF samples.

One of the advantages of this study is that it includes 17 children with long-term and intensive follow-up, with a mean of 3 examinations per child and a total of 58 FLT measurements. Therefore, FLT results could be compared to the clinical, histological and imaging results at each time point. FLT was found to be reliable, reproducible, and highly correlated to the clinical course, treatment, and outcome.

According to the literature, about one-third of children with medulloblastoma have metastatic disease within the CNS at diagnosis^6^. The presence of CNS metastases is assessed in two ways: prior to surgery by MRI of the whole craniospinal axis and approximately 2 weeks after surgery by cytologic study of CSF samples. Lumbar puncture to collect CSF cannot be performed before surgery because of the risk of brain stem herniation. In patients with metastatic disease at diagnosis, treatment is usually upgraded to include a higher dose of craniospinal irradiation and possibly autologous bone marrow transplantation following high-dose chemotherapy.

MRI is limited in this setting because micrometastases appear as equivocal cord irregularities and may be confused with blood vessels and other structures. The cytologic study, performed when the post-surgery edema has decreased but prior to the initiation of chemo/radiotherapy, consists of microscopic examination of the CSF for appearance and staining characteristics of cells^26^. Its accuracy depends largely on the quality of the sample and the experience of the cytopathologist who must, in equivocal cases, decide if the findings are pathological. Furthermore, studies have shown that M1 disease (presence of positive cells in the CSF) is also a poor prognostic marker, even in children with normal-appearing imaging studies, and is treated with high-risk treatment protocols^27, 28^ similar to those used for overt metastasis^4^.

Thus, better means to rapidly and accurately identify malignant spread of pediatric brain tumours are needed. Accuracy is essential because cells in the CSF at 2 weeks after surgery may be indicative of postoperative complications, including infection and inflammation, as well as true metastatic spread. The failure to correctly differentiate between malignant and non-malignant cells may lead to over- or under-treatment^5^. Over-treatment predominantly involves the dose of radiation. The higher radiation dose of the high-risk as opposed to the average-risk protocol (36.0 vs 23.4 GY CSI for patients older than 3 years) makes an enormous difference in outcome in terms of long-term side effects, including poor cognitive functioning, short stature, and hearing impairment. At the same time, under-treatment may pose a risk of reduced survival, since salvage of relapsed patients is low, particularly after prior radiation.

In the present study, we identified 3 different FLT populations that differentiated among cells of different types and at different stages. Despite the variability in each slide and the wide FLT range, the majority of the cells from each patient (as calculated by the median) were significantly indicative of the clinical stage (non-metastatic/metastatic) and response to treatment. In one of our patients (no. 3, Fig. 2a), the cytology results were interpreted as inflammatory cells only, and treatment was given according to the average-risk protocol for non-metastatic disease. However, FLIM showed that the cells were probably malignant. Since FLIM has not yet been validated as a clinical tool, it cannot yet be used to guide clinical decision-making. This patient is currently 21 months from diagnosis and only 4 months since completing treatment, and he is under close follow-up. In another patient who had an ATRT (no. 10, Fig. 2f), the cells in the CSF at diagnosis were considered to be metastatic on cytological study and probably on FLIM as well (median FLT 3.86 ns). It is interesting the FLT in the CSF reached a nadir at 7 months from diagnosis (median 1.4 ns) while the patient was still on treatment but started to increase (median 2.78 ns) at completion of treatment 11 months after diagnosis, with what was interpreted as a normal MRI scan. Just 4 months later, a spinal metastasis was detected on MRI. At biopsy, the metastatic lesion had a similar FLT (median 2.75 ns) to that of the CSF 5 months previously. We speculate in retrospect that the patient was not in remission when we stopped treatment. It is possible that after treatment, cells do not reach the long FLT seen at diagnosis, and the rise in FLT may be the more sensitive sign of impending relapse. Clearly, we need more samples from patients who eventually relapse (only one, no. 10, in the present study). As we analyze more samples, it is likely that a clearer cutoff value for FLT for malignant spread will be available for clinical use.

The persistence of malignant cells after treatment is equally worrying and may indicate a need to upscale treatment. Similarly, detection of relapse at an earlier stage, when the disease is still at the microscopic level, may theoretically improve the patient’s chances with therapies targeted at minimal residual disease burden.

Recently, Ferreira-Facio *et al*.^29^ described the application of flow cytometry, for the first time, for the differentiation of brain tumours. Flow cytometry is currently commonly used in haemato-oncology for the analysis of tumour, bone marrow, blood, and other body fluids. Specific markers can identify haematological vs. non-haematological cells and even distinguish among different solid tumours of similar embryonal origin, such as neuroblastomas and primitive neuroectodermal tumours. However, in their study of brain tumours, Ferreira-Facio *et al*.^29^ did not evaluate the accuracy of flow cytometry for differentiating metastatic from non-metastatic cells in the CSF of children with brain tumours, or for clinical staging, as we have for FLIM.

When monitoring cell functionality, cytometrists more frequently use fluorescence intensity rather than FLT. However, unlike direct fluorescent intensity measurements, which are strongly dependent on non-relevant variables (optical factors, light source instability, etc.), FLT is intrinsic, thereby allowing for more reliable comparisons of data obtained from different experiments and different laboratories. High FLT values in cells (6 ns vs. 2.6 ns in the present study) may point to a significant restriction on mobility of the probe imposed by its intracellular microenvironment^25^. The FLT of free DAPI is 2 ns, and the FLT of DAPI bound to DNA in normal cells is 2.7 ns^26^. We recently reported shorter (1 ns) as well as longer (6 ns) FLT values of DAPI in B-cells from leukemia patients^30^. There are several possible causes of such restriction, namely, structural properties (e.g., caging and “wobbling-in-cone”)^31^, binding properties (covalent bonding, dipole-dipole interactions, or interactions of higher orders of charge arrangement), or simply solvent viscosity. The present results point to the viscosity of the fluorophore-hosting area as the culprit in the FLT alterations. This is in line with our previous findings of a clear correlation of FLT and processes of the peripheral blood mononuclear cell response to mitogens, as well as to antigens and phorbol esters^32^ and the contraction cycle of cardiac cells^33, 34^.

The results of our FLT studies were nearly all in accordance with the cytology results. CSF samples were taken only when clinically indicated; in some patients, when the disease was clearly metastatic, no samples were obtained. Our results yielded a statistically significant difference between metastatic and local disease, and high correlations with the clinical observations and outcome were noted over the follow-up period. With a larger cohort, multidimensional scaling analysis as well as a machine-learning approach could be performed in order to quantify the power of FLIM technology and detect metastatic disease in the early stages. To overcome the variability of measurements for the same patient, we measured as many cells as possible per slide and calculated the median. Within each cell-source group (A-F), except for the non-malignant inflammatory group (group G), there was wide variability among the patients, but we found the median values of each group to be representative and useful for comparing the groups at different stages of treatment.

In the future, it may be possible to distinguish metastatic from localised disease by measuring circulating DNA in the blood or CSF^35–37^. There are already some reports describing the assessment of cell-free DNA in the CSF as a tumour marker^38, 39^. These techniques hold great promise for the future.

At present, in the absence of supersensitive methods to detect minute quantities of tumour in DNA or RNA in blood, the FLIM system is a promising auxiliary tool for the early and reliable detection of metastatic spread in the CSF of children with malignant brain tumours.

## Methods

### Setting, patients, and sample collection

The study was conducted at Schneider Children’s Medical Center of Israel. The study design adhered to the tenets of the Declaration of Helsinki and was approved by the Schneider Children’s Medical Center of Israel and national review boards of the Israel Ministry of Health before its initiation. Informed consent was obtained from all parents. The anonymous CSF samples were obtained under separate institutional review board approval and exempted from consent.

Tumour samples were collected at primary surgery. CSF samples were collected at least 2 weeks after primary surgery, during the course of diagnostic lumbar puncture performed prior to chemo/radiotherapy. Additional CSF samples were collected after administration of chemotherapy, radiotherapy, or both as part of the routine clinical follow-up. No lumbar punctures were performed for research purposes. Demographic, imaging, and treatment data were collected from the medical files.

Control samples of CSF with inflammatory cells derived from patients with non-oncological, infectious diseases attending the same hospital during the same period were collected from the hospital’s microbiology laboratory.

### Preparation of Tumour and CSF cells

A tissue cut of up to 10 mg from tumour samples was subjected to gentle pipetation with 1 ml pipete, in order to separate the cells. Tumour and CSF samples were centrifuged at 600 × g for 7 min. The supernatant fluid was gently removed, and 500 μl of PBS solution were added to the sediment cells. These steps were then repeated. After the second centrifuge, the supernatant fluid was gently removed, and 10 μl of a solution containing methanol and acetic acid (3:2) were added to the cells, followed by rapid transfer of 20 μl of the cells to the center of a slide. After 7 min, the slide was transferred through an ethanol gradient (70%, 85%, 100% for 2 min each). Two drops of NucBlue® Fixed Cell Stain ReadyProbes® Reagent (Thermo Fisher Scientific, Waltham, MA, catalog no. R37606) were diluted in 1 ml phosphate-buffered saline, and 200 μl of this 4′,6-diamidino-2-phenylindole (DAPI) solution were added to the slide. After 5 min, unincorporated DAPI solution was removed, and 10 μl of ProLong® Gold Antifade Mountant Reagent (Thermo Fisher Scientific, catalog no. P36930) were added.

### FLIM measurements and analysis

The FLIM experiments were performed using an Olympus IX-81 inverted microscope with a 10X, NA = 0.4 objective (Olympus, Japan). The images were acquired with a Lambert Instruments (LI) system (Groningen, The Netherlands) consisting of 3 modulated LED excitation sources at 403 nm, 468 nm, and 537 nm, a LI^2^CAM MD intensified CCD camera with modulated image intensifier for detection of the fluorescence images, a modulation signal generator/power supply unit, computer, and the LI-FLIM software package for complete system control, image acquisition, and FLT calculation. The excitation source of 403 nm was set to a repetition frequency between 40 and 60 MHz for heterogeneous samples; the emission wavelengths were 420 nm and above. In slides rich with cells, 20 to 106 cells were randomly selected and measured. When there were fewer than 10 cells, all were measured. Each measurement was repeated twice. Median FLT was calculated per slide. As shown in previous studies, the FLT of free DAPI is 2 ns, and the FLT of DAPI in normal cells is 2.7 ns^26^. As a control experiment, cells from the same patients were measured without DAPI staining and below magnification of MCP GAIN of 750 ± 50 V. Under these conditions, no fluorescence intensity was detected. Above 750 ± 50 V, weak fluorescence intensity signals can be extracted, with average FLT of 1.57 ± 0.64 ns (data not shown). Except for this phenomenon, no autofluorescence was observed. In our experiments, we used magnification in the range of 620-730 V.

It is well known from the literature that the FLT of DAPI has a heterogeneous distribution^30^. In our study, the FLT measurements of DAPI bound to DNA in tumour cells were divided into subgroups, as described in a previous study on the use of FLIM to classify B-cell chronic lymphocytic leukemia^30^. The FLT of the DNA binder DAPI found in healthy cells, τ = 2.66 ± 0.12 nsec (range 2.3–2.92 nsec), correlated with the FLT of DAPI in the control group. The three groups with an abnormal value were classified as short+, prolonged, and prolonged + according to their relative proximity to the normal group: τ ≪ norm (τ < 1.8 nsec), τ > norm (3 nsec < τ < 4.5 nsec), and τ ≫ norm (τ > 4.5 nsec), respectively. FLIM measurements revealed that values in the range of 1.8 nsec < τ < 2.2 nsec were related to free DAPI (not bound to DNA) and therefore were not part of the analysis (2 ± 0.12 ns). The FLT value was calculated by extracting the phase FLT (τ_φ_) and the modulation FLT (τ_m_), yielding a total single FLT value.

### Statistical analysis

Boxplots and density plots were generated using R statistical language^40^. Patients were divided into different groups by time from diagnosis, therapy stage, and disease status. The FLT values were normalised before merging in order to prevent bias due to differences in cell number between slides. Due to the high variability of the FLT measured per slide, median and median absolute deviation were calculated per slide and per group. Median absolute deviation is insensitive to the presence of outliers, unlike the standard mean/standard deviation combination. Wilcoxon signed rank test was used to test the significance of differences in FLT distribution. We compared the control group (G) against each of the other groups, separately. We also compared each group against the CSF metastatic, pre-therapy group (B). Hierarchical clustering was performed using R framework by applying a 1-correlation distance measure and average-linkage clustering.

### Ethics approval and consent to participate

The study was approved by the national and institutional ethics committees. Informed consent was obtained from the parents of all children. The anonymous samples of the CSF obtained for the control group were exempted from consent.
